# Ecosystem Functions Connecting Contributions from Ecosystem Services to Human Wellbeing in a Mangrove System in Northern Taiwan

**DOI:** 10.3390/ijerph120606542

**Published:** 2015-06-09

**Authors:** Hwey-Lian Hsieh, Hsing-Juh Lin, Shang-Shu Shih, Chang-Po Chen

**Affiliations:** 1 Biodiversity Research Center, Academia Sinica, Taipei 115, Taiwan; E-Mails: zohl@gate.sinica.edu.tw (H.-L.H.); hjlin@dragon.nchu.edu.tw (H.-J.L.); 2 Department of Life Sciences and Research Center for Global Change Biology, National Chung Hsing University, Taichung 402, Taiwan; 3 Hydrotech Research Institute, National Taiwan University, Taipei 106, Taiwan; E-Mail: uptreeshih@ntu.edu.tw

**Keywords:** mangrove ecosystem, ecosystem functions, ecosystem services, human wellbeing, component connection network

## Abstract

The present study examined a mangrove ecosystem in northern Taiwan to determine how the various components of ecosystem function, ecosystem services and human wellbeing are connected. The overall contributions of mangrove services to specific components of human wellbeing were also assessed. A network was developed and evaluated by an expert panel consisting of hydrologists, ecologists, and experts in the field of culture, landscape or architecture. The results showed that supporting habitats was the most important function to human wellbeing, while water quality, habitable climate, air quality, recreational opportunities, and knowledge systems were services that were strongly linked to human welfare. Security of continuous supply of services appeared to be the key to a comfortable life. From a bottom-up and top-down perspective, knowledge systems (a service) were most supported by ecosystem functions, while the security of continuous supply of services (wellbeing) had affected the most services. In addition, the overall benefits of mangrove services to human prosperity concentrated on mental health, security of continuous supply of services, and physical health.

## 1. Introduction

It has been well documented that the mangrove ecosystem, one of the most important ecosystems in the biosphere, provides broad, valuable services to human societies in tropical and subtropical areas as well as to the entire biosphere [1,2,3]. The interactions between ecosystem services and human socio-economics are bidirectional [4,5]. Mangrove trees alone do not create a biologically diverse ecosystem, nor do they make the ecosystem function in a healthy manner. In addition to mangrove vegetation, other essential components of a mangrove ecosystem include mudflats, tidal waterways, and shallow water areas. As a result, this system hosts diverse aquatic and terrestrial fauna and flora [6,7]. Another crucial component to mangrove ecosystems is circulating water, which connects all parts of the system [8]. Water acts to incorporate the mangrove swamp as a subsystem of river or coastal ecosystems, depending on whether the mangrove is distributed in an estuary or along the coast. These unique distributional settings create substantially distinct ecosystem service values. For instance, coastal mangroves contribute heavily to wave attenuation [2,9]. However, this may not be the case for estuarine or riverine mangroves, where mangrove expansion increases the flooding risk to adjacent residents by reducing the water conveyance area and thereby lengthening the inundation period [10]. This means that the most important mangrove ecosystem services depend on locality, composition, and the presence of people.

The interactions between hydrological and biological components affect the functions of the mangrove system, which are related to services that ultimately support human wellbeing. However, numerous documents indicate that humans have altered mangrove functionality by placing enormous demands on various mangrove services. These supply-demand couplings reflect the fact that ecosystem functions and services are connected to human wellbeing in both bottom-up and top-down directions. These three levels—function, service, and wellbeing—exist in a hierarchy and are composed of a number of components. Each component varies in the magnitude of its contribution to the next higher or lower level. Therefore, a better understanding of the interactions between the natural driving forces that shape mangrove services and the socio-economic uses of mangrove services can benefit humans. More importantly, finding the relationships between mangrove services and human welfare can assist stakeholders in the formulation of sustainable action plans that support mangrove ecosystems. Previous studies have addressed mangrove ecosystems’ natural drivers [11], functions [7,12], services [2], monetary valuations [3], ecological restoration [2], and management and adaptation strategies in response to threats from climate changes [13]. Framework development procedures and a simplified network, with respect to the linkages between ecosystem functions, ecosystem services, and community’s wellbeing, were illustrated for rainforests of South East Queensland, Australia [14]. The present study uses these framework development procedures to advance our knowledge of the complexity of interactions between a mangrove ecosystem and resultant benefits to human wellbeing. A mangrove system located close to an urban area was chosen as an ideal case study site.

The Danshuei River ecosystem in northern Taiwan has sustained the Taipei metropolis for centuries. It is renowned for its estuarine mangrove nature reserves, which are protected by Taiwan’s Culture Heritage Preservation Law [15]. These reserves are vegetated by a monoculture of the mangrove plant *Kandelia obovata*, which is capable of tolerating the unusual cold in the Danshuei estuary (e.g., temperatures below 10 °C for 7 days) [16,17]. These reserves appear to contain the largest population of *Kandelia obovata* in the Northern Hemisphere [18]. At one time, the reserves thrived and supported a diverse and abundant array of fauna and flora. Migratory shorebirds and waterfowl included the globally threatened Chinese Egret *Egretta eulophotes*, the Black-faced Spoonbill *Platalea minor* and the abundant Common Teal *Anas crecca*. The latter had a maximum record of 10,000 counts [19]. Fisheries of *Scylla* mud crabs and blunt nose snake eels were also plentiful [20]. Besides the mangrove trees, the sedge *Cyperus malaccensis* was an important harvested plant and the common reed *Phragmites communis* was widely distributed [10]. However, continuous seeding and expansion of the dense mangrove trees have resulted in a series of alterations to both the hydrological regime and biotic communities within the reserves. Flood risk increased, which resulted in tree birds replacing shorebirds as the dominant avian species, and benthic diatom production decreased, which shifted the structure of benthic communities from polychaetes and amphipods to crabs [10,21,22]. The overgrowth led to the local extinction of the sedge *Cyperus malaccensis* [23,24] and confined the distribution of the Taiwan endemic fiddler crab *Uca formosensis* (H.L. Hsieh, personal observations). Recently, a study on trade-offs between reducing flood risks and storing carbon stocks revealed that the removal of up to 20% of vegetated mangrove areas appeared to optimize the cost-benefit ratio of mangrove management in the Danshuei estuary [25].

Given the uniqueness of the plant, *Kandelia obovate*, and the conflicting values of services resulting from its over-growth, as well as the adverse effects of its interactions with hydrology on biodiversity, a broadly based study is warranted. A better understanding of the connections between different mangrove ecosystem functions and services, and human wellbeing, will allow us to grasp how the services of these mangrove reserves relate to human wellbeing. Specifically, the purpose of the present study is to unravel and describe the underlying mechanisms within mangrove ecosystems that affect how interdependent features evolve through hierarchical bottom-up and top-down processes. We do this by linking basic ecosystem functions to the utilization of ecosystem services and the fulfillment of human needs. Using the mangrove ecosystem of Danshuei River as a case study, this purpose can be achieved by assessing two objectives: (1) defining and describing the bottom-up and top-down connections between mangrove ecosystem functions, ecosystem services, and human wellbeing; and (2) synthesizing the overall bottom-up contributions of the mangrove’s services to human wellbeing.

## 2. Materials and Methods

### 2.1. Study Area

**Figure 1:**
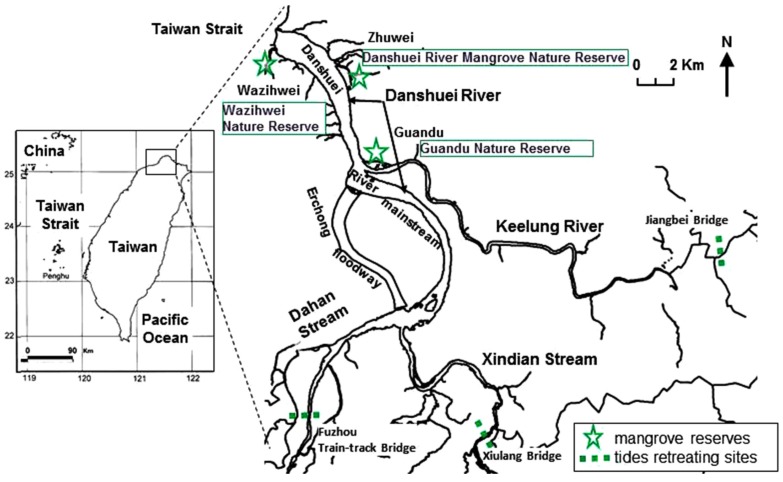
Map of the study area in the Danshuei River estuary. Asterisks: three mangrove reserves; dashed lines: sites where tides begin to retreat.

The study area, the Danshuei River, is located close to Taipei City in northern Taiwan (Figure 1). The River is fed by three main tributaries: Dahan Stream, Xindian Stream and the Keelung River and has a watershed area of approximately 3216 km^2^. The combined length of the main stream of each tributary is approximately 81 km, and the waters flow through the most densely populated districts of the Taipei and New Taipei metropolis. This region houses approximately 3.83 million people. The estuary is influenced by a semidiurnal tidal regime with a mean tidal amplitude of 2.17 m and a maximum amplitude of up to 3 m during spring tides in the lower estuary [26]. Salinity fluctuates from 5 to 33 and is often negligible in the upper estuarine reaches. Dissolved oxygen concentrations range from a low of 2.24 at the Guandu site to a high of 6.51 mg·L^−1^ at the Zhuwei site, which indicates the importance of tidal action in reducing pollution effluent from the urban areas in the upper reaches [27]. Mangrove forests are primarily distributed in the lower estuary and consist almost exclusively of *Kandelia obovata*. The three mangrove protected areas are the Wazihwei Nature Reserve, the Danshuei River Mangrove Nature Reserve and the Guandu Nature Reserve, with 2.87, 2.04, and 3.04 stands per m^2^, respectively [28,29] (Figure 1).

### 2.2. Interconnection Network Analyses of Functions, Services and Wellbeing

Ecosystem functions are defined as “the capacity of natural processes and components to provide goods and services that satisfy human needs, directly or indirectly” [30]. Ecosystem services are “the benefits people obtain from an ecosystem” [4]. Like other ecosystems, mangroves provide four categories of functions and services: Provision, Regulation, Support, and Culture Functions. Except for Support, the other three are well recognized categories of ecosystem services that contribute to human wellbeing. Human wellbeing can be measured using five categories: Existence, Health, Security, Good Social Relations, and Freedom of Choice and Action [4,31]. These categories were derived from previous research on the valuations of ecosystem services [4,14,30,31,32], and we adopted the frameworks associated with the categories, with some modifications to itemize the components of each category to fit the case of the Danshuei mangrove ecosystem. This itemization was based on a series of discussions among ecologists, hydrologists, and cultural researchers in Taiwan.

A total of 33, 28, and 15 components were identified for the Danshuei mangrove functions, services, and contributions to human wellbeing, respectively (Table 1). A network connection model of links between the mangrove system’s functions and services, and services and wellbeing, was constructed based on the aforementioned levels and categories, identified components from the frameworks and approaches used by Maynard *et al.* [14], and an input and output model commonly used in industrial interrelationship analyses [33]. The network consisted of three subsets and was assessed from four perspectives. The three subsets were: the links between the mangrove system and the function levels, between the function and service levels, and between the service and human wellbeing levels. The first perspective was a bottom-up view that specified which components at each level formed important links between the three subsets of the network. The second perspective traced connections among the most important components at the service level using both bottom-up and top-down relationships. The third perspective evaluated the most frequent links in the top-down direction between wellbeing and services. The fourth perspective focused on overall contributions of mangrove services to the components of human wellbeing.

**Figure 2:**
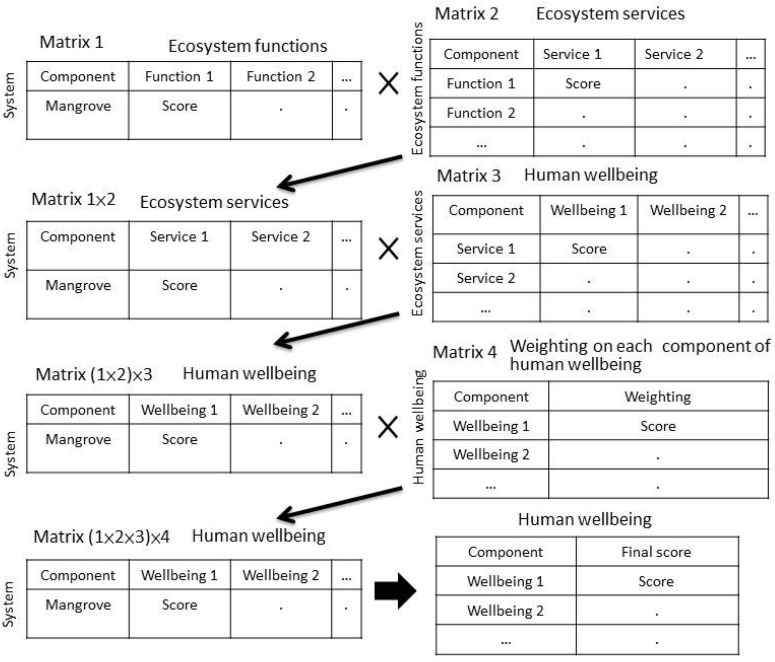
Matrices used by expert panel to score and prioritize components of ecosystem functions, services, and human wellbeing provided by the mangrove system in the Danshuei Estuary ecosystem. Notations x and arrows depict the procedures of multiplications between matrices and proceed to yield scores on the resultant matrices; x: multiplication sign. See Table 1 for the component contents and Materials and Methods session for procedure details.

The relative magnitudes of connections or relationships among components at each of the three levels (ecosystem functions, ecosystem services and human wellbeing) of the Danshuei River mangrove system were determined by first formulating three matrices—Matrix 1, 2, and 3—and then having an expert panel evaluate those matrices using simple scores (Figure 2). When scoring the corresponding matrix-pairs, the higher the score obtained by a given component pair, the stronger the connection or contribution present in that pair. The same procedures were performed to evaluate the strength of connections between the mangrove system and its functions, ecosystem functions and ecosystem services, and ecosystem services and human wellbeing.

The relative contribution of overall services of the mangrove system to each component of human wellbeing was also analyzed simultaneously using an additional matrix, Matrix 4, in which each component of human wellbeing was weighted by the same expert panel (Figure 2). To analyze the data, the scores given for corresponding matrix-pairs were multiplied through three consecutive multiplication steps (Figure 2). The higher the final scores obtained for each component of human wellbeing, the greater the potential contributions of the mangrove system to that given welfare component. The context of each of the four matrices is described as follows:

Matrix 1: Connections or contributions from mangrove system to each component of ecosystem function;
Matrix 2: Connections or contributions from each component of ecosystem function to each component of ecosystem services;
Matrix 3: Connections or contributions from each component of ecosystem services to each component of human wellbeing;
Matrix 4: Expert weighting for each component of human wellbeing.

For Matrices 1, 2, and 3, the scores were classified into 6 grades. The scoring criteria was: score 0—no connection, score 1—weak and indirect connection, score 2—weakly indirect connection, score 3—strongly indirect connection, score 4—weakly direct connection, and score 5—strong and direct connection. For Matrix 4, the scores were classified into 5 levels as follows: score 1—very unimportant contribution, score 2—unimportant contribution, score 3—moderate contribution, score 4—important contribution, and score 5—very important contribution.

**Table 1:** The components of ecosystem functions, services and human wellbeing assessed for the Danshuei estuarine mangrove system. In the first two columns, the letter notations shown in parentheses indicate the Category to which each constituent belongs, R: Regulation, P: Provision, S: Support, and C: Culture. In the third column, the letter notations in parentheses represent E: Existence, H: Health, SE: Security, GSR: Good Social Relations, and FCA: Freedom of Choice and Action. Category definitions adapt those used in [4,31], and Maynard *et al.* [14].

No.	Ecosystem Functions	Ecosystem Services	Human Wellbeing
1	Climate regulation (R)	Water quality (R)	Breathing (E)
2	Microclimate stabilization (R)	Habitable climate (R)	Drinking (E)
3	Coastline stabilization (R)	Air quality (R)	Nutrition (E)
4	Flood regulation (R)	Arable land (R)	Shelter (E)
5	Storm protection and Tsunami impact mitigation (R)	Buffering against extremes (R)	Physical health (H)
6	Water recycling (R)	Pollination (R)	Mental health (H)
7	Underground water replenishment (R)	Reduction of pests and diseases (R)	Security of continuous supply of services (SE)
8	Soil retention (R)	Productive soils (R)	Security of person (SE)
9	Nutrient regulation (R)	Noise abatement (R)	Security of health (SE)
10	Waste treatment and assimilation (R)	Food (P)	Security of access to services (SE)
11	Pollination (R)	Water for consumption (P)	Security of property(SE)
12	Biological control (R)	Building materials and Fibers (P)	Family cohesion (GSR)
13	Barrier effect of vegetation (R)	Fuels (P)	Community and social cohesion (GSR)
14	Carbon sequestration (R)	Genetic resources (P)	Social and economic freedom(FCA)
15	Supporting other ecosystems (S)	Bio-chemicals, Medicines and Pharmaceuticals (P)	Self-actualization (FCA)
16	Biodiversity maintenance (S)	Ornamental resources (P)	
17	Nutrient retention (S)	Transport infrastructure (P)	
18	Nutrient cycling (S)	Recreational opportunities (C)	
19	Primary productivity (S)	Healing landscape (C)	
20	Soil formation (S)	Aesthetic values (C)	
21	Agricultural resources (P)	Knowledge systems (C)	
22	Fishery resources (P)	Iconic landscapes (C)	
23	Wood industry resources (P)	Iconic species (C)	
24	Hunting resources (P)	Cultural diversity (C)	
25	Energy resources (P)	Spiritual and religious values (C)	
26	Water supply (P)	Inspiration (C)	
27	Genetic resources (P)	Affecting on social interactions (C)	
28	Supporting habitats (P)	Sense of place belonging (C)	
29	Pharmacological resources (P)		
30	Holy landscape (C)		
31	Symbolic landscape (C)		
32	Healing landscape (C)		
33	Residential landscape (C)		

The final scores given by the experts for each paired connection in Matrices 1, 2, and 3 were calculated as Fuzzy values following the Fuzzy Delphi method [34]. To identify which connections or contributions were important, Fuzzy values located in the top 25% were taken as threshold values. A given connection or contribution with a Fuzzy value greater than this threshold was considered important and was retained to construct the network of links among the mangrove system, ecosystem functions, ecosystem services and eventually, human wellbeing. The final scores given to each component of human wellbeing in Matrix 4 were the mathematic means averaged from all experts’ scores.

The Fuzzy values were calculated as follows:

Fuzzy value = (a + b + m)/3

where a: the maximum value from the original experts’ scores; b: the minimum value from the original experts’ scores; m: geometric mean of the original experts’ scores.

The important lineages of connection from bottom-up and/or top-down directions among functions, services, and human wellbeing were further analyzed. In the bottom-up lineages, the components at the function level with scores in top 10% (*i.e.*, ≥4.17) were selected and linked to the service and human wellbeing levels. Those with lower scores but with the highest score, in terms of contribution to the service level, were also selected for bottom-up linking. In both the top-down and bottom-up lineages at the service level and the top-down lineages at the wellbeing level, the components with the most frequent links were traced. A total of nine connection lineages were identified.

The expert panel consisted of 20 researchers with specialties in three fields: hydrology; ecology; and a cultural, landscape, or architecture field. Each field had six, eight, and six experts, respectively. Evaluation questionnaires were delivered to the expert panel in February 2012.

## 3. Results

**Figure 3:**
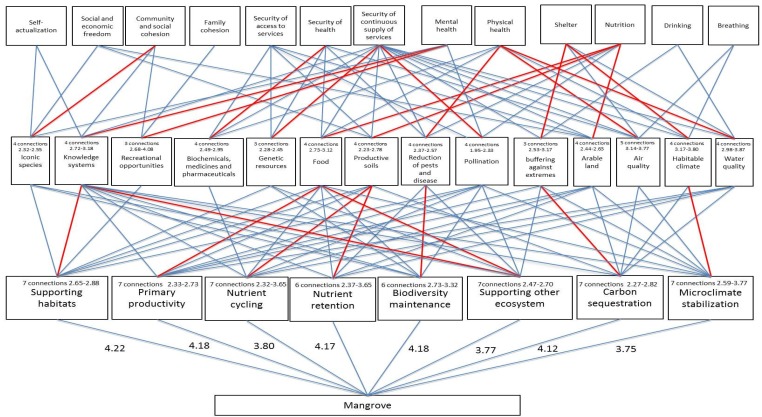
The connection network for the components of ecosystem functions, ecosystem services and human wellbeing provided by the Danshuei River mangrove system. Red lines depict paired connections with the highest scores, thus reflecting the most important relationships. Blue lines show the connections with Fuzzy values in the top 25%. Numerical numbers inserted in the top lines within boxes indicate the numbers of connections provided by ecosystem functions to ecosystem services and those by services to wellbeing. Numerical numbers following the word “connection” in the top line are Fuzzy values with ranges from the top 25% to maximum values.

The connection network for the components of ecosystem functions, ecosystem services and human wellbeing provided by the Danshuei River mangrove system is shown in Figure 3. The network reveals the important components at each level, the connections for different bottom-up and top-down lineages, as well as the overall contributions of mangrove services to the humankind’s prosperity.

### 3.1. The Important Components of Functions, Services and Wellbeing

Among the eight most noticeable functions identified for mangroves (out of 33), supporting habitats (Provision category) was identified as the most important function (score 4.22), while primary productivity, biodiversity maintenance, and nutrient retention (which fell into the Support category) were also important but had slightly lower scores (4.17 to 4.18). The remaining four functions provided by the mangroves were microhabitat stabilization, carbon sequestration, supporting other ecosystems, and nutrient cycling (scores ranging from 3.75 to 4.12, Figure 3).

Fourteen out of 28 service components were identified as the most important mangrove services. These services were: water quality, habitable climate, air quality, arable land, buffering against extremes, pollination, reduction of pests and diseases, productive soils, food, genetic resources, bio-chemicals, medicines and pharmaceuticals, recreational opportunities, knowledge systems, and iconic species (Figure 3).

Thirteen out of 15 human wellbeing components were evaluated as important. These included breathing, drinking, nutrition, shelter, physical health, mental health, security of continuous supply of services, security of health, security of access to services, family cohesion, community and social cohesion, social and economic freedom, and self-actualization (Figure 3).

### 3.2. Bottom-up Connections between Functions and Services

In general, each of the eight components at the function level performed six to seven connections to the mangrove services level as shown in red and blue lines in Figure 3. The component microhabitat stabilization function in the Regulation Function category (scores 2.59 to 3.77) and the components nutrient cycling, nutrient retention and biodiversity maintenance functions in the Support Function category (scores 2.32 to 3.65) exhibited strong connections to the service level. Among seven services derived from the mangrove microhabitat stabilization function, habitable climate service was the most important as it gained the highest score of 3.77 compared to the rest of 53 connections (a total of 54 connections) between the function and service levels.

### 3.3. Bottom-Up Connections between Services and Human Wellbeing

Each of the 14 components at the mangrove service level appeared to be associated with three to five components of human wellbeing. Water quality, habitable climate, and air quality (Regulatory Service category) contributed greatly to human wellbeing (scores 2.98 to 3.87), while recreational opportunities and knowledge systems (Culture Service category) also had strong connections to human wellbeing (scores 2.68 to 4.08). The recreational opportunities service appeared to be the most important service offered by mangroves to human wellbeing, as this service had the highest score (up to 4.08). In addition, it was closely related to the mental health. With respect to the habitable climate and water quality services, both services played important roles in shelter and physical health wellbeing, respectively (Figure 3). It is worth mentioning that iconic species, a cultural component derived from mangrove services, was closely associated with community and social cohesion.

### 3.4. The Important Lineages of Bottom-up and/or Top-down Connections between Functions, Services and Human Wellbeing

Nine strong connection lineages were notable and deserved further illustration (Figure 3). These included six bottom-up, two bi-directional, and one top-down network. The bottom-up networks were as follows: (1) supporting habitats (Function) greatly contributed to positive mental health (Human Wellbeing) through knowledge systems (Service, a high score of 3.18); (2) microclimate stabilization (Function) closely linked to the habitable climate (Service), which supported the shelter (Human Wellbeing); (3) biodiversity maintenance (Function) played an important role in the reduction of pests and disease (Service) and in turn, strengthened an important part of human’s physical health (Human wellbeing); (4) nutrient retention (Function) noticeably contributed to the human nutrition (Human wellbeing) via the regulation of productive soils (Service); (5) primary productivity (Function) contributed to human nutrition (Human wellbeing) through the provision of food (Service); and (6) supporting habitats (Function) appeared to correlate with the recreational opportunities (Service). The recreational opportunity service made a pronounced contribution to mental health wellbeing (gaining the highest score of 4.08 among service components), although the linking magnitude of this pairing showed a greater variation than that for other pairings.

The two bi-directional networks, where the ecosystem services had the greatest impact on both ecosystem function and human wellbeing were: (1) knowledge systems in the cultural services category and (2) food in the provisional services category. These two components appeared to have the most intense networks of relationships between ecosystem function and service, as there were eight and seven connections to the function level, respectively. Knowledge systems further contributed to mental health, while food met the human nutrition need. Among the top-down links within this setting, the service of knowledge systems appeared to be strongly associated with the functions of supporting other ecosystems and supporting habitats, whereas food had important relationships to the functions of supporting other ecosystems and primary productivity.

The one top-down network was related to the security of continuous supply of services in the Security Wellbeing category. It was heavily dependent on mangrove services, including eight regulatory, three provisional, and one cultural component. The most important connections were bound to genetic resources and pollination services.

### 3.5. Overall Bottom-Up Contributions of Mangrove Services to Human Wellbeing

The relative importance of each component of human wellbeing as relates to the provision of the mangrove services is listed in Table 2. Among the 15 wellbeing components listed, mental health in the Health Wellbeing category was the most important wellbeing attribute (score 76,021) derived from mangrove services. Next to mental health, ecosystem services contributed greatly to four other wellbeing components in the categories of Security, Health, Freedom of Choice and Action, and Good Social Relations. Those components were security of continuous supply of services, physical health, self-actualization, and community and social cohesion, respectively (scores 61,277 to 70,450).

**Table 2:** Relative importance of human wellbeing components provided by the mangrove system in the Danshuei estuary. Score values are the final scores produced in the Matrix (1 × 2 × 3) × 4 shown in Figure 2.

Category	Components	Score Value
Existence	Breathing	45,465
	Drinking	52,581
	Nutrition	47,001
	Shelter	58,050
Health	Physical health	64,563
	Mental health	76,021
Security	Security of continuous supply of services	70,450
	Security of person	49,541
	Security of health	54,065
	Security of access to services	52,181
	Security of property	32,784
Good social relations	Family cohesion	56,109
	Community and social cohesion	61,277
Freedom of choice and action	Social and economic freedom	55,406
	Self-actualization	62,857

## 4. Discussion

Using the matrix connection approach, the present study has led to new insights into the interdependent associations among mangrove ecosystem functions, ecosystem services and human wellbeing. In addition, the present study clearly reveals that mangrove ecosystems, as in the case of the Danshuei estuary, are comprised of complicated networks of relationships. These networks link important components in a bottom-up direction, from basic ecosystem functions to ecosystem services, and transfer a great deal of mangrove services to benefit human wellbeing. These networks also illustrate the top-down relationships of human wellbeing to ecosystem services, implying the transmission of substantial influences to the functional level. These interconnected features are addressed, interpreted and discussed below.

### 4.1. Insights from the Networking of Mangrove Ecosystem Functions, Ecosystem Services and Human Wellbeing

Using a matrix networking approach with an input and output model is evidently useful in evaluating the links between the functions, services, and human wellbeing associated with mangrove ecosystems. The generalities that make this approach work provide new insights into the interdependent connections among many components at different levels of a complicated network. These insights include:

(1) Multidisciplinary assessment: The network had to be constructed by a panel of experts. Those experts had different areas of concern, including hydrology, ecology, culture, landscape design and architecture. Through a cross- and multi-disciplinary assessment, the network produced represents the integration of various viewpoints that led to the development of a rich field of Regulation, Provision, Support and Culture categories at the mangrove function and service levels. More importantly, the network describes the even more complicated Health, Security, Good Social Relations, and Freedom of Choice and Action categories at the human wellbeing level. As a result, this network achieves consensus in the identification of important components at each level.

(2) Implication of bottom-up and top-down connections: The network illustrates the bi-directional interactions among mangrove functions and services and human wellbeing. The bottom-up connections reflect the support provided by the mangrove ecosystem on which mankind’s welfare depends. Such links help in the identification of which service components are most important to a human society at any given time. When a service or services decrease in quality or are lost, an improvement or restoration practice at the function level can be made to improve those services, and management plans should be adjusted accordingly. Top-down connections reflect the influential consequences of mankind’s actions on services and functions, which result in the sustainability or collapse of mangroves. The downward links help to guide humans’ actions on wise use of mangrove services. With these bidirectional connections, the network described here demonstrates the extensive yet interdependent associations between mankind’s prosperity and mangrove functions and services.

(3) Adaptability of the network: The network exhibits broad adaptability. The components of each level (function, service, or human wellbeing) were adopted from those described in several renowned studies on the bases of global ecosystems [4,14]. However, which components of which level are most important, and therefore highlighted with the network, depend on the ecosystem, locality, components, and people. Therefore, the network possesses both general and special features. For example, the network discussed here is applicable to other countries for developing management plans that can benefit their people with the continuous supply of services from mangrove ecosystems.

### 4.2. Overall Bottom-Up Contributions from Mangrove Services to Human Wellbeing

The results of the present study have shown that mental health, security of continuous supply of services, and physical health are three most important components provided by the study mangroves to ensure mankind’s satisfactory existence (see Table 2). Health refers to an individual who has the basic material for a good life [4], and, more than this, who is capable of coping with changes in external environments and in one’s self [14] while acting as a productive member of society [35]. Within the service context of the mangroves, use-services such as water quality, habitable climate, air quality, food, bio-chemicals, medicines and pharmaceuticals are basic determinants of humans’ physical health, whereas non-use services such as recreational opportunities and knowledge systems are absolutely indispensable to humans’ mental health (see Figure 3). The coupled relationships between services and wellbeing components reflect that mangrove ecosystem services are attached to human nourishment (e.g., food), shelter (e.g., habitable climate), disease prevention (e.g., chemicals, medicines such as benzoxazoline, triterpenoid saponins and quinones [36]), and clean water and air accessibility. These services also relate to inspirational, aesthetic and recreational experiences (e.g., knowledge systems and recreational opportunities).

Security of continuous supply of services is one of the Security category’s attributes. This attribute refers to an individual who is able to secure access to natural and other resources, and to perceive safety of person and possessions. It also refers to an individual who is able to cope with natural and human-made disasters [4]. The aforementioned service context benefits not only mankind’s health, but also its security. Mangrove services including arable land, buffering against extremes, pollination, reduction of pests and disease, productive soils, and genetic resources, which are all tightly associated with the necessity for security, as these services ensure secure access to natural and knowledge systems resources (e.g., arable land, productive soils, genetic resources, pollination), personal and personal possession safety (e.g., reduction of pests and disease), and security from natural and anthropogenic disasters (e.g., buffering against extremes).

Self-actualization in the Freedom of Choice and Action category is the fourth most important wellbeing component impacted by the services of mangroves (see Table 2). This attribute refers to an individual who has the opportunity to achieve what the individual values doing and being [4]. Self-actualization is the most advanced demand in Maslow’s hierarchy of needs for life. It requires an individual to become their best self [37,38]. Among the services provided by mangroves, knowledge systems and iconic species appear to meet the need of self-actualization (see Figure 3). As self-actualization is often expressed in the creativity, innovation and identity of art, science and technology, the knowledge systems service and iconic species service likely play key roles in nurturing and advancing personal creativity, innovation and identity. These two services were also found to be extremely important in contributing to self-actualization in the study that developed an ecosystem services framework for Australia’s South East Queensland [38].

Community and social cohesion in the Good Social Relations category also depends heavily on mangrove services. This wellbeing attribute refers to the relationships and working links between groups, cultures and communities. This basic need is satisfied when an individual feels belonging to a larger, friendly, supportive group with people who show mutual respect [38]. Among services derived from the mangroves, recreational opportunities, knowledge systems, and iconic species contribute greatly to community and social cohesion, because these services are needed for a happy life. The recreational opportunities provided by mangroves can promote personal social interactions with people who have the same or similar passions for appreciating, taking care of and restoring nature. These mangrove swamps support knowledge systems that facilitate learning, understanding, and the development of trust and support for one another, which in turn allow people to share mangrove services in an equal, fair and continuous manner.

Iconic species in estuarine mangroves include the viviparous seedling mangrove *Kandelia obovata* and the fiddler crab. They are easily recognizable and emblematic. They have unique appearances that can capture the imagination of the public. For instance, the propagules of *K. obovata* are nicknamed “water pen” in Chinese, and fiddler crabs are commonly known as “tide-beckoning” crabs. The Danshuei mangrove case and others [38] have shown that iconic species often provide the public a means of drawing consensus and engaging in conservation. Iconic species also play a significant role in ecotourism, which benefits local livelihoods. As a result, the services derived from iconic species living in mangroves can promote good relations within communities and society in general.

### 4.3. Highlights of Some Important Linkages of Mangrove Functions and Services to Human Wellbeing

There are several strong bottom-up links connecting mangrove functions to human wellbeing through mangrove services. These connections relate to specific components and are as follows:

(1) Contributions of supporting habitats, microclimate stabilization, and biodiversity maintenance to human health: A healthy mangrove system possesses diverse habitats, which in turn, support biodiversity [1,2,22,39]. Understanding the science-based knowledge related to the formation of habitats and biodiversity can satisfy mankind’s curiosity about nature. Sensing biodiversity also fulfills human’s need to consume natural aesthetics [2]. These fascinating experiences contribute to mental health. Greater biodiversity and maintenance of native species act as a buffer against pest and disease incidences in croplands, terrestrial and aquatic ecosystems, and human populations [40,41]. Therefore, a reduction in pests and diseases (a service) derived from the function of biodiversity maintenance appears to strengthen an important part of human physical health. Mangrove ecosystems can cool down and humidify the local atmosphere [42]. Mangrove areas also serve as windbreaks, thus protecting local residences [43]. The services generated from microclimate stabilization (a function) contribute heavily to shelter requirements (wellbeing), which is a prerequisite for humans’ existence.

(2) Contributions of primary productivity and nutrient retention to human nutrition: Mangrove ecosystems are some of the most productive ecosystems in the world because of their high photosynthetic rates [1,2]. They are also renowned as nutrient reservoirs [2,12]. The nutrients make up sediments, which are occupied by a variety of microbes, primary producers and detritus that form the foundational food pools [44] needed for productive shellfisheries and fisheries [2]. This benefits mankind’s need for nutrition through the provision of food (a service).

(3) Contribution of recreational opportunities to mental health: The number of visitors to mangroves increases because of the attractiveness of the magnificent aesthetics and ecological uniqueness of mangroves [8,45]. In the surrounding Danshuei River estuary mangrove reserves, there are walking paths, bike trails and green corridors, which make sightseeing, relaxation, and bird or crab watching accessible (authors’ personal observations). Educational centers established nearby those reserves have engaged in nature-based education and promote community participation in wetland protection [46,47,48]. These educational and recreational opportunities benefit the participants’ mental health. Substantial evidence links natural environments, such as mangroves, with good physical and mental health [49].

(4) Contribution of pollination and genetic resources to the security of continuous supply of services: Honey and bees-wax production is of national and local importance and is a renewable resource [50]. Coincidentally, pollination performed by those insects also has advantages for commercial crops and other forests. In addition, the genetic resources of mangrove ecosystems are tremendously rich and include the numerous varieties of microbes, fauna and flora living there. Those genes and genetic information are very useful for animal and plant breeding as well as biotechnology [4]. Therefore, pollination and genetic resources services can fulfill mankind’s need for a continuous and reliable supply of products from mangrove ecosystems.

### 4.4. Implication of Supporting Habitats in Mangrove Management

Habitat support appears to be the most important function provided by the Danshuei River mangrove ecosystem. However, mangrove forests are among the most threatened ecosystems in the world [2]. Loss of habitat diversity in Taiwan’s estuaries has also been dramatic [22]. Given that the human wellbeing demands are often responsible for top-down, adverse consequences on mangroves [2], humans should be able to develop management plans to reduce those threats. In order to appropriately manage the situations recorded globally and locally [2,22], an inventory is needed to identify what kinds of habitats have been lost or are degraded and where and why such deterioration occurs.

## 5. Conclusions

The network generated from the studied mangrove ecosystem reveals many of the underlying connections that link mangrove services to human wellbeing and ensure mankind’s prosperity. The network also demonstrates how mankind’s demand on mangrove services can result in substantial influences on basic mangrove functions. We used a matrix connection approach to derive the network, which possesses the characteristics of multidisciplinary assessment, bottom-up and top-down linking, and adaptability. The present study suggests a potential new approach for the development of management plans that can foster both local and global mangrove ecosystem sustainability, as well as the sustainability of other types of ecosystems.
